# Supplementary material for: Gene Therapy with Voretigene Neparvovec Improves Vision and Partially Restores Electrophysiological Function in Pre-School Children with Leber Congenital Amaurosis
Source: Biomedicines. 2022 Dec 30;11(1):103. doi: 10.3390/biomedicines11010103 (PMC9855623; doi:10.3390/biomedicines11010103)
Supplement: Supplementary file 1 [file biomedicines-11-00103-s001.zip › Suppl. Figure S1. 30Hz Flicker-ERG of patient 2 after gene therapy with voretigene neparvovec.pdf]

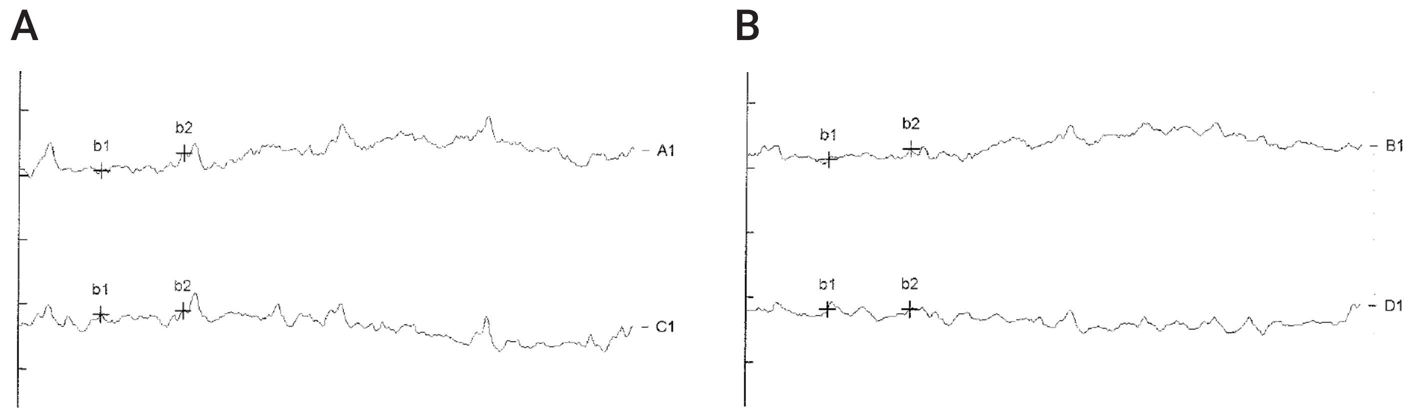

SUPPL. FIGURE S1. 30HZ FLICKER-ERG OF PATIENT 2 AFTER GENE THERAPY WITH VORETIGENE NEPARVOVEC. (A) shows reproducible Flicker-ERG responses in the right eye of a 3-year-old male (P2) recorded six months after subretinal gene therapy. In contrast to the right eye, no clear evidence of 30Hz flicker responses could be detected in the left eye (B). Unfortunately, a repeat of the ERG could not be performed yet because both the patient and his parents disaproved further exams. Both Flicker ERGs are shown at a magnification of 10X.
