# Supplementary material for: Gene Therapy with Voretigene Neparvovec Improves Vision and Partially Restores Electrophysiological Function in Pre-School Children with Leber Congenital Amaurosis
Source: Biomedicines. 2022 Dec 30;11(1):103. doi: 10.3390/biomedicines11010103 (PMC9855623; doi:10.3390/biomedicines11010103)
Supplement: Supplementary file 1 [file biomedicines-11-00103-s001.zip › Suppl. Figure S2. Goldmann visual fields of patient 1 and patient 3 after gene therapy.pdf]

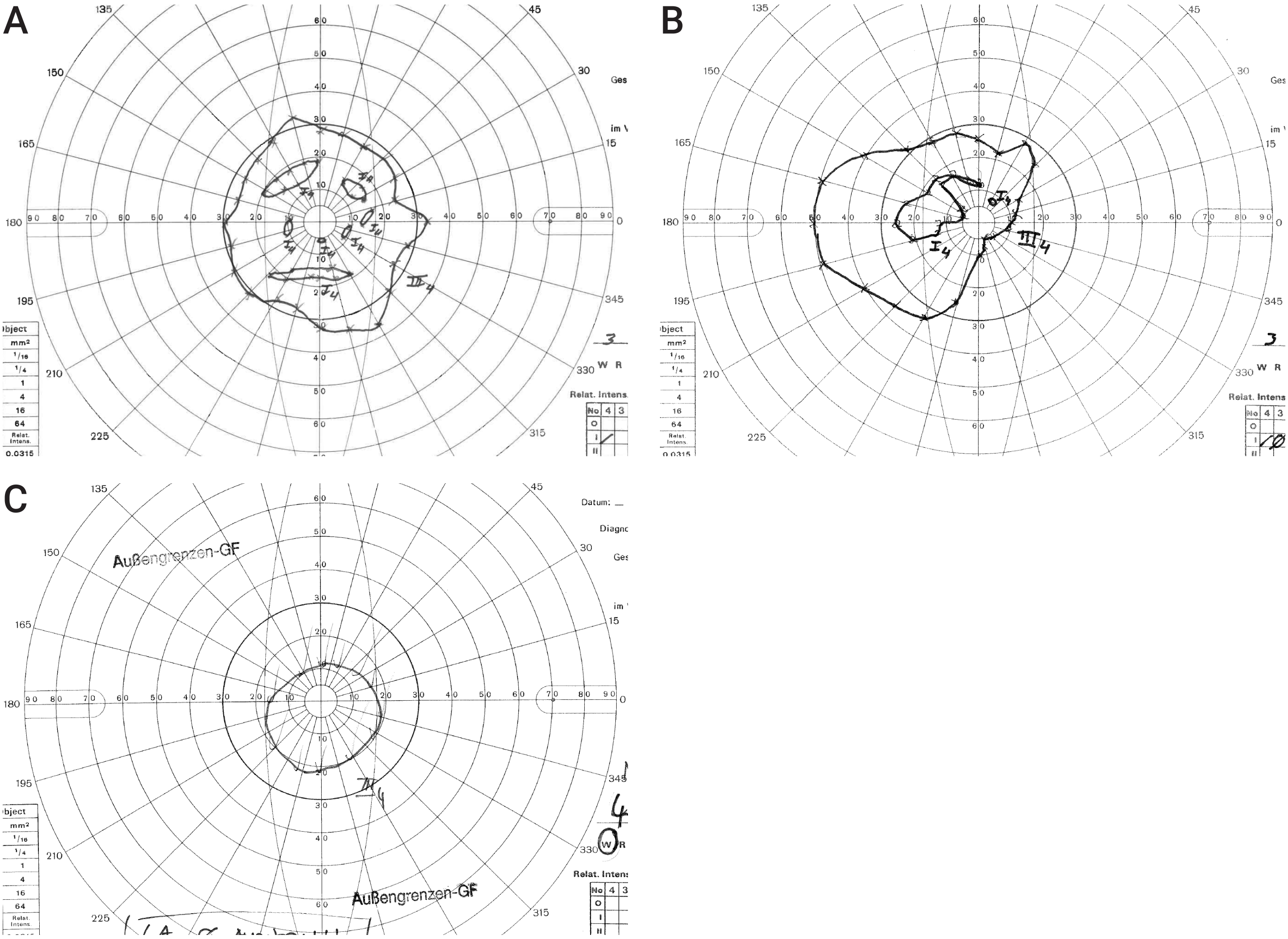

SUPPL. FIGURE 2. GOLDMANN VISUAL FIELDS OF PATIENT 1 AND PATIENT 3 AFTER GENE THERAPY. Upper Panel (A-B) shows visual fields of a 4-year-old child (P1) approximately 2 years (OD 21months, OS 23months) after subretinal gene therapy. Reliable visual field testing was possible showing multiple visual field islands for target I_4 between 5°-20° and outer limits between 25°-37° (III_4) in the right eye (A). Visual field testing of the left eye (B) revealed visual field islands for target I_4 between 5°-26° and outer limits between 5°-50° (III_4). Notably, visual field testing before treatment could not be performed due to severely impaired vision. (C) shows visual field of a 5-year-old child (P3). Similarly to P1, preoperative visual field testing could not be performed due to severely impaired vision and nystagmus. Six months after subretinal gene therapy, outer visual field limits between 10°-22° could be determined using the III_4 target despite of postoperative complication with retinal detachment and re-attachment surgery involving silicon oil implantation. At time of assessment, the left eye of P3 was not yet treated which explains the missing data. The illustrated isopters correspond to the aforementioned targets and represent contour lines of the hill of vision.
