# Supplementary material for: Gene Therapy with Voretigene Neparvovec Improves Vision and Partially Restores Electrophysiological Function in Pre-School Children with Leber Congenital Amaurosis
Source: Biomedicines. 2022 Dec 30;11(1):103. doi: 10.3390/biomedicines11010103 (PMC9855623; doi:10.3390/biomedicines11010103)
Supplement: Supplementary file 1 [file biomedicines-11-00103-s001.zip › Suppl. Figure S3A. VFQ25 visualization_Part2 individual responses.pdf]

A

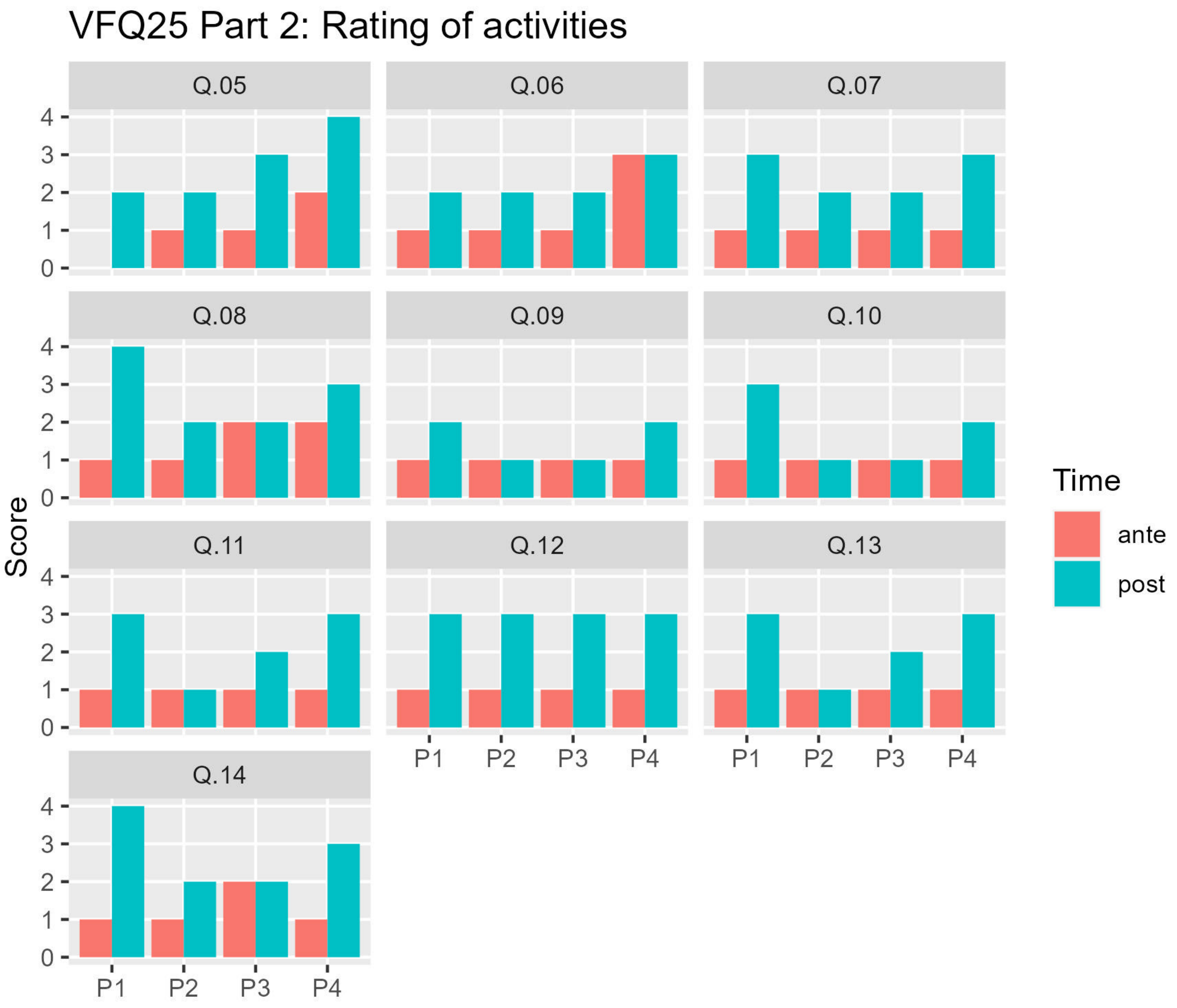


Q05. How much difficulty do you have reading ordinary print in newspapers?
Q06. How much difficulty do you have doing work or hobbies that require you to see well up close, such as cooking, sewing, fixing things around the house, or using hand tools?
Q07. Because of your eyesight, how much difficulty do you have finding something on a crowded shelf?
Q08. How much difficulty do you have reading street signs or the names of stores?
Q09. Because of your eyesight, how much difficulty do you have going down steps, stairs, or curbs in dim light or at night?
Q10. Because of your eyesight, how much difficulty do you have noticing objects off to the side while you are walking along?
Q11. Because of your eyesight, how much difficulty do you have seeing how people react to things you say?
Q12. Because of your eyesight, how much difficulty do you have picking out and matching your own clothes?
Q13. Because of your eyesight, how much difficulty do you have visiting with people in their homes, at parties, or in restaurants ?
Q14. Because of your eyesight, how much difficulty do you have going out to see movies, plays, or sports events?

**SUPPL. FIGURE S3A. VISION-RELATED BEHAVIORAL AND PSYCHOMETRIC CHANGES AFTER GENE THERAPY.** Indi-vidual responses to questions on certain activities that require good eyesight (VFQ25 Part2) before and after gene therapy with voretigene neparvovec are shown. Responses before therapy (ante) are represented by red columns, whereas the turquoise columns represent responses given 6 months after gene therapy (post). Individual scores for each patient and item ranged from 0 (representing the worst possible answer) to 4 (most positive answer as one would expect from a nor-mally sighted person). Illustrated are the scores of each item for all treated patients (P1-P4).
