# Supplementary material for: Gene Therapy with Voretigene Neparvovec Improves Vision and Partially Restores Electrophysiological Function in Pre-School Children with Leber Congenital Amaurosis
Source: Biomedicines. 2022 Dec 30;11(1):103. doi: 10.3390/biomedicines11010103 (PMC9855623; doi:10.3390/biomedicines11010103)
Supplement: Supplementary file 1 [file biomedicines-11-00103-s001.zip › Suppl. Figure S3B. VFQ25 visualization_Part3 individual responses.pdf]

B

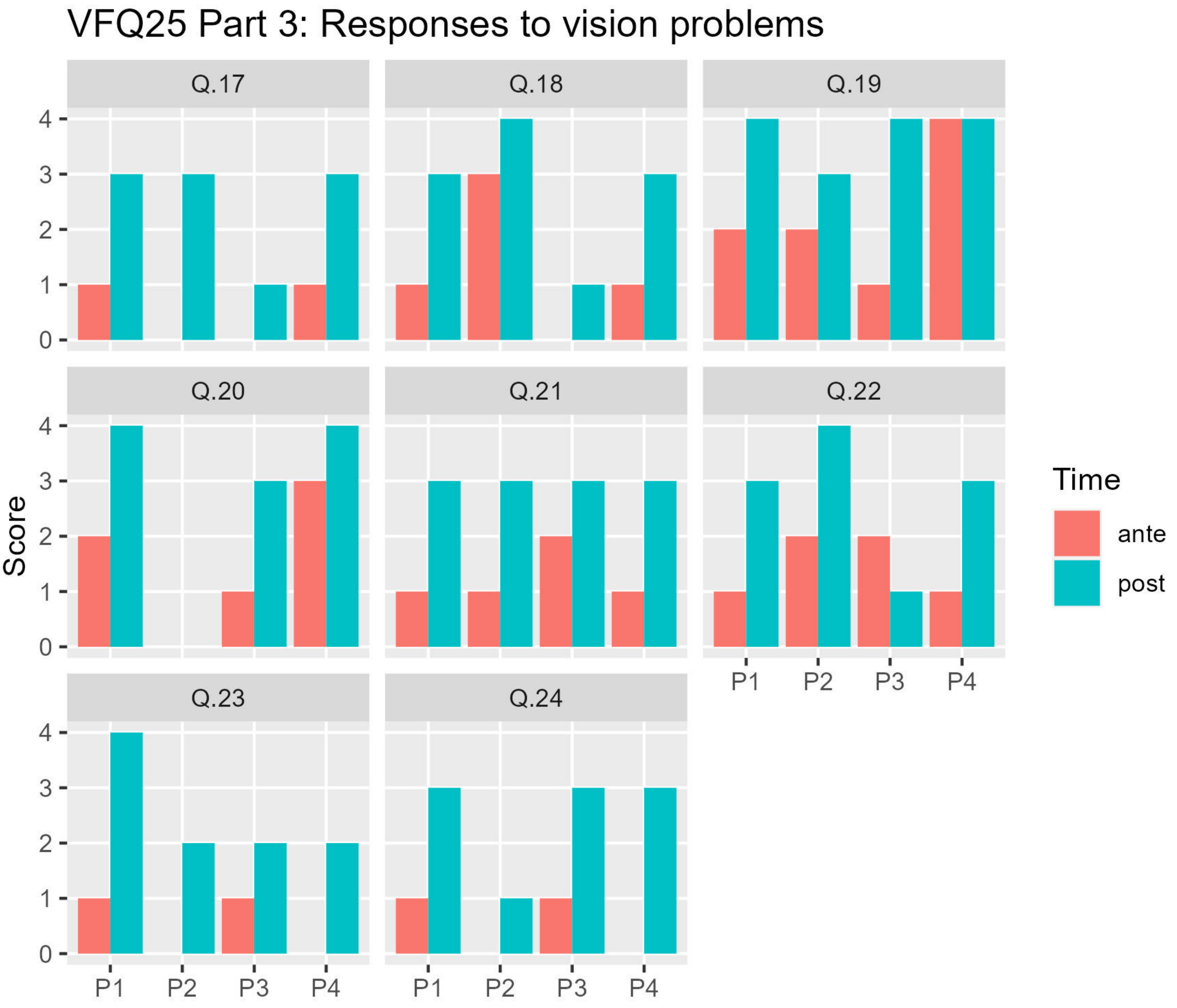

Q17. Do you accomplish less than you would like because of your vision?
Q18. Are you limited in how long you can work or do other activities because of your vision?
Q19. How much does pain or discomfort in or around your eyes, e.g. burning, itching, aching keep you from doing what you'd like to be doing?
Q20. I stay at home most of the time because of my eyesight
Q21. I feel frustrated a lot of the time because of my eyesight
Q22. I have much less control over what I do, because of my eyesight
Q23. Because of my eyesight, I have to rely too much on what other people tell me
Q24. I need a lot of help from others because of my eyesight

SUPPL. FIGURE S3B. VISION-RELATED BEHAVIORAL AND PSYCHOMETRIC CHANGES AFTER GENE THERAPY. Indi-vidual responses to questions about things whose performance may be affected by vision (VFQ25 Part3) before and after gene therapy with voretigene neparvovec are shown. Responses before therapy (ante) are represented by red columns, whereas the turquoise columns represent responses given 6 months after gene therapy (post). Individual scores for each patient and item ranged from 0 (representing the worst possible answer) to 4 (most positive answer as one would expect from a normally sighted person). Illustrated are the scores of each item for all treated patients (P1-P4).
