# Supplementary material for: Gene Therapy with Voretigene Neparvovec Improves Vision and Partially Restores Electrophysiological Function in Pre-School Children with Leber Congenital Amaurosis
Source: Biomedicines. 2022 Dec 30;11(1):103. doi: 10.3390/biomedicines11010103 (PMC9855623; doi:10.3390/biomedicines11010103)
Supplement: Supplementary file 1 [file biomedicines-11-00103-s001.zip › Suppl. Table S1. Overview of performed examinations and timeline showing available data for each timepoint after gene therapy.pdf]

Table S1. Overview of performed examinations and timeline showing available data for each timepoint after gene therapy.

Timeline (months after surgery)				1	2	3	4	6	8	12	14	15	20	21	22	23	30	32
Patient	Examination	Eye	Baseline / before gene therapy															
P1	BCVA (LogMAR)*	OD<br>OS	1.0<br>1.3	0.8<br>1.1		0.8<br>0.8		1.0<br>0.9		0.6<br>x		x<br>0.7		0.5<br>x		x<br>0.6	0.5<br>x	x<br>0.6
	BCVA (LogMAR)*	OD<br>OS	BCVA change to baseline	-0.2<br>-0.2		-0.2<br>-0.5		0<br>-0.4		-0.4<br>x		x<br>-0.6		-0.5<br>x		x<br>-0.7	-0.5<br>x	x<br>-0.7
	ERG (30Hz)	OD<br>OS	✓<br>✓	-		-		-		-		-		✓<br>x		x<br>✓	✓<br>x	x<br>✓
	FST (dB)	OD<br>OS	n.a. **	-		-		-		-		-		-		-	n.a.◆◆<br>n.a.◆◆	n.a.◆◆<br>n.a.◆◆
	Visual Field		n.a. **	-		-		-		-		-		✓<br>(OD)		✓<br>(OS)	-	-
	OCT	OD<br>OS	✓<br>✓	✓<br>✓		✓<br>✓		✓<br>✓		✓<br>x		x<br>✓		✓<br>x		x<br>✓	✓<br>x	x<br>✓
	Autofluorescence	OD<br>OS	✓<br>✓	✓<br>✓		✓<br>✓		✓<br>✓		✓<br>x		x<br>✓		✓<br>x		x<br>✓	✓<br>x	x<br>✓
	Wide-angle fundus imaging	OD<br>OS	✓<br>✓	✓<br>✓		✓<br>✓		✓<br>✓		✓<br>x		x<br>✓		✓<br>x		x<br>✓	✓<br>x	x<br>✓
Timeline (months after surgery)				1	2	3	4	6	8	12	14	15	20	21	22	23	25	27
P2	BCVA (LogMAR)*	OD<br>OS	1.1<br>(OU near)	0.8<br>0.7		0.6<br>0.7		0.6<br>0.7		0.9<br>0.7	0.7<br>x		x<br>0.7		0.9<br>x		x<br>0.5	0.9<br>x
	BCVA (LogMAR)*	OD<br>OS	BCVA change to baseline	-0.3<br>-0.4		-0.5<br>-0.4		-0.5<br>-0.4		-0.2<br>-0.4	-0.4<br>x		x<br>-0.4		-0.2<br>x		x<br>-0.6	-0.2<br>x
	ERG (30Hz)	OD	n.a. **	-		-		✓		-	-		n.a. ◆		n.a. ◆		n.a. ◆	n.a. ◆

		OS						✓					n.a. ◆		n.a. ◆		n.a. ◆	n.a. ◆
	FST (dB)	OU	n.a. **	-		-		n.a. **		-	-		n.a. **		n.a. **		n.a. **	n.a. **
	Visual Field		n.a. **	-		-		n.a. **		-	-		n.a. **		n.a. **		n.a. **	n.a. **
	OCT	OD	OD	✓		✓		✓		✓	✓		x		✓		x	✓
		OS	OS	✓		✓		✓		✓	x		✓		x		✓	x
	Autofluorescence	OD	✓	✓		✓		✓		✓	✓		x		✓		x	✓
		OS	✓	✓		✓		✓		✓	x		✓		x		✓	x
	Wide-angle fundus imaging	OD	✓	✓		✓		✓		✓	✓		x		✓		x	✓
		OS	✓	✓		✓		✓		✓	x		✓		x		✓	x
Timeline (months after surgery)				1	2	3	4	6	8	12								
P3	BCVA (LogMAR)*	OD	1.2	1.3		1.3		0.8		1.2								
		OS	1.2	1.2		x		1.0		x								
	BCVA (LogMAR)*	OD	BCVA change to baseline	0.1		0.1		-0.4		0								
		OS		0		x		-0.2		x								
	ERG (30Hz)	OD	✓	-	-	-	-	x	-									
		OS	✓					✓										
	FST (dB)	OD	n.a. **	-	-	-	-	x	-37.41	-58.20								
		OS						-32.12	x	x								
	Visual Field		n.a. **	-	-	-	-	✓ (OD)										
	OCT	OD	✓	✓		✓		✓		✓								
		OS	✓	✓		x		✓		x								
	Autofluorescence	OD	✓	✓		✓		✓		✓								
		OS	✓	✓		x		✓		x								
	Wide-angle fundus imaging	OD	✓	✓		✓		✓		✓								
		OS	✓	✓		x		✓		x								
Timeline (months after surgery)				1	2	3	4	6	8									
P4	BCVA (LogMAR)*	OD	0.5	0.4	0.4	0.3	0.5	0.2	x									
		OS	0.7	0.5	x	0.5	x	0.4	0									
	BCVA (LogMAR)*	OD	BCVA change to baseline	-0.1	-0.1	-0.2	0	-0.3	x									
		OS		-0.2	x	-0.2	x	-0.3	-0.7									

	**ERG (30Hz)**	**OD**<br>**OS**	✓<br>✓	**-**	**-**	**-**	**-**	**n.a.** ◆<br>**n.a.** ◆	**-**
	**FST (dB)**	**OD**<br>**OS**	**-09.55**<br>**-10.76**	**-**	**-**	**-**	**-**	**-38.05**<br>**-66.25**	**-**
	**Visual Field**		✓ **(OU)**	**-**	**-**	**-**	**-**	✓<br>**(OD)**	✓<br>**(OS)**
	**OCT**	**OD**<br>**OS**	✓<br>✓	✓<br>✓	✓<br>**x**	✓<br>✓	✓<br>**x**	✓<br>✓	**x**<br>✓
	**Autofluorescence**	**OD**<br>**OS**	✓<br>✓	✓<br>✓	✓<br>**x**	✓<br>✓	✓<br>**x**	✓<br>✓	**x**<br>✓
	**Wide-angle fundus imaging**	**OD**<br>**OS**	✓<br>✓	✓<br>✓	✓<br>**x**	✓<br>✓	✓<br>**x**	✓<br>✓	**x**<br>✓

* visual acuity was assessed in decimal and converted in LogMAR. In P1, visual acuity before treatment was assessed using LEA symbols and in the postoperative course using Landolt C rings (single optotypes); in P2, LEA symbols were used during the entire observation period; P3 was assessed using an ETDRS number chart and assessment of P4 was performed using Landolt C rings (single optotypes) during the entire observation period

** due to demanding set-up, visual impairment, and young age no measurements could be obtained

✓ examination was performed

◆ examination could not be carried out due to non-compliance (patient refused exam)

◆◆ poor data quality, no valid values could be recorded due to reduced compliance of the patient

x due to the interval between each surgery, values for both eyes are not always available for each time point (x = missing data)

“ - “ = examination was not performed because it was not scheduled as part of the routine check-up
